# Beyond Traditional Antimicrobials: A *Caenorhabditis elegans* Model for Discovery of Novel Anti-infectives

**DOI:** 10.3389/fmicb.2016.01956

**Published:** 2016-12-02

**Authors:** Cin Kong, Su-Anne Eng, Mei-Perng Lim, Sheila Nathan

**Affiliations:** School of Biosciences and Biotechnology, Faculty of Science and Technology, Universiti Kebangsaan MalaysiaBangi, Malaysia

**Keywords:** *Caenorhabditis elegans*, antimicrobials, anti-virulence, immunomodulator, antimicrobial peptides

## Abstract

The spread of antibiotic resistance amongst bacterial pathogens has led to an urgent need for new antimicrobial compounds with novel modes of action that minimize the potential for drug resistance. To date, the development of new antimicrobial drugs is still lagging far behind the rising demand, partly owing to the absence of an effective screening platform. Over the last decade, the nematode *Caenorhabditis elegans* has been incorporated as a whole animal screening platform for antimicrobials. This development is taking advantage of the vast knowledge on worm physiology and how it interacts with bacterial and fungal pathogens. In addition to allowing for *in vivo* selection of compounds with promising anti-microbial properties, the whole animal *C. elegans* screening system has also permitted the discovery of novel compounds targeting infection processes that only manifest during the course of pathogen infection of the host. Another advantage of using *C. elegans* in the search for new antimicrobials is that the worm itself is a source of potential antimicrobial effectors which constitute part of its immune defense response to thwart infections. This has led to the evaluation of effector molecules, particularly antimicrobial proteins and peptides (APPs), as candidates for further development as therapeutic agents. In this review, we provide an overview on use of the *C. elegans* model for identification of novel anti-infectives. We highlight some highly potential lead compounds obtained from *C. elegans*-based screens, particularly those that target bacterial virulence or host defense to eradicate infections, a mechanism distinct from the action of conventional antibiotics. We also review the prospect of using *C. elegans* APPs as an antimicrobial strategy to treat infections.

## Introduction

It is undeniable that antibiotics have had an enormous impact on global human health by drastically reducing infection-associated mortality. Nonetheless, the abuse and uncontrolled use of antibiotics has resulted in the emergence and spread of resistant bacteria. Whilst resistance to antibiotics has escalated steadily, the number of new antimicrobial drugs approved, especially those with novel modes of action, continues to decline (Boucher et al., 2013). Among the vast number of Gram-positive and Gram-negative bacteria, the ‘ESKAPE’ group of pathogens (*Enterococcus faecium*, *Staphylococcus aureus*, *Klebsiella pneumoniae*, *Acinetobacter baumannii*, *Pseudomonas aeruginosa*, and *Enterobacter* species) represent the most common antibiotic-resistance pathogens (Pendleton et al., 2013). The emergence of variant pathogens such as the New Delhi metallo-ß-lactamase 1 (NDM-1)-producing Enterobacteriaceae strains (Kumarasamy et al., 2010) and colistin-resistant *Escherichia coli* harboring the *mcr-1* gene (Liu et al., 2016; McGann et al., 2016) have exacerbated the situation and further underscores the importance of new innovative anti-infective strategies to minimize the spread of drug resistance. A major obstacle in the identification of effective anti-infective therapies is the absence of efficient *in vivo* screening platforms. Since the first introduction of *Caenorhabditis elegans* to study developmental biology, the worm has proven to be a versatile host model for elucidating molecular and cellular aspects of various infectious diseases. *C. elegans* has been adopted as an *in vivo* infection model to dissect the intricate host–pathogen interaction and the evolutionarily conserved mechanisms employed by pathogens to infect and kill the host (Ermolaeva and Schumacher, 2014; Cohen and Troemel, 2015). The descriptions of an expanding list of bacterial and fungal pathogen infections in *C. elegans* (Marsh and May, 2012) have led to the use of worms as a whole organism system for antimicrobial and anti-infective drug discovery. This review will assess the use of *C. elegans* as a platform for the discovery of novel anti-infectives or antimicrobial compounds that target bacterial virulence or host immune responses to attenuate infections. We also highlight the potential of *C. elegans* as a rich source of antimicrobial proteins and peptides (APPs), key components of innate immunity.

## *C. elegans* Platform For Anti-Infectives Discovery: Why And How?

The increasing popularity of *C. elegans* as a host model is attributed to its small (∼1 mm) and simple anatomy, short generation time, high fecundity, fully sequenced genome (∼100 Mb) and the relatively easy and inexpensive maintenance. Moreover, working with the worm offers great advantage as many resources of genetic and genomic knowledge and techniques are available to facilitate experimental manipulation (Antoshechkin and Sternberg, 2007). For example, knocking down worm genes can easily be achieved through RNA interference (RNAi) simply by feeding the worms with bacteria harboring a plasmid engineered to express double-stranded RNA (dsRNA) targeting the gene of interest. Two RNAi clone libraries covering almost 94% of the 20 000 genes in *C. elegans* are available and this allows genome-wide RNAi screens to be performed (Kamath and Ahringer, 2003; Rual et al., 2004). Furthermore, over 3000 phenotypically defined mutant strains are available from the Caenorhabditis Genetics Centre. In addition, the worm is transparent, allowing for *in vivo* monitoring of cells and the visualization of fluorescently tagged bacterial and host genes and proteins throughout the entire course of an experiment. The nematode defense system is highly homologous to at least three mammalian conserved innate immunity signaling pathways which are crucial for defense against pathogens; the p38 Mitogen-Activated Protein Kinase (p38 MAPK) pathway (Troemel et al., 2006), the Insulin/Growth Factor-1 (IIS) pathway (Garsin et al., 2003) and the Transforming Growth Factor-β (TGF-β) pathway (Mallo et al., 2002). Studies have also indicated that many of the virulence factors involved in the killing of worms were also required for the pathogenesis of mammals (Tan et al., 1999; Sifri et al., 2003) and hence, over the last decade, the utility of the worm has also been extended to facilitate novel antimicrobial drug discovery and development (Ewbank and Zugasti, 2011; Arvanitis et al., 2013). Taken together, these advantages make *C. elegans* an ideal model organism in various aspects of biology.

The traditional pipeline for antimicrobial drug discovery usually begins with *in vitro* screening of test compounds followed by subjecting potential hits to *in vivo* animal testing (Brown and Wright, 2016). However, these potential compounds often end up exhibiting poor pharmacokinetic activities and/or are highly toxic when tested *in vivo* in animals. Another approach is to directly determine the *in vivo* effects of the test compounds in conventional mammalian models but this approach presents with serious limitations, including high cost, laborious and time-consuming procedures and ethical constraints (Arvanitis et al., 2013). While mammalian models have been utilized to study potentially new drug leads, screening of a large number of compounds is prohibited with these models. Both these approaches have shortcomings and cause significant delay in antimicrobial discovery. To overcome these limitations, an alternative method is to conduct the initial testing of compounds in the *C. elegans* whole animal system. Exposing worms to both pathogen and test compounds and subsequently monitoring worm survival throughout the assay can be easily performed as an antimicrobial screen of test compounds in *C. elegans*. Compounds with potential antimicrobial activity will contribute to prolonged infected worm lifespan when compared to the untreated infected worms. Screening of compounds with the *C. elegans* whole animal model also allows preliminary assessment of drug toxicity as worms treated with these compounds will show similar or decreased survival when compared to the untreated control (Moy et al., 2009). A further advantage of the *C. elegans* model is the self-fertilization of hermaphrodite worms which allows for cost effective rapid cultivation of a large population of worms for drug testing without any of the ethical constraints normally encountered when working with rodents and primates. The worm is also amenable to fully automated high-throughput screening whereby 100s of animals can be systematically dispensed into wells of standard 96- and 384- well plates permitting the screen of larger compound libraries, the incorporation of fluorescent dye to ease scoring of dead and alive worms as well as the use of sophisticated hardware and software for image capturing and data analysis (Wahlby et al., 2012; Conery et al., 2014). To increase the efficiency of the high-throughput screen, improvements to the automated worm sorting and transfer technologies, imaging and software for data analysis are constantly being undertaken (O’Reilly et al., 2014).

Another significant advantage of using *C. elegans* in the search for new antimicrobial compounds is that, as an *in vivo* model, it allows for the detection of compounds that may not directly target the pathogen viability but instead, may alter pathogen virulence or enhance the host immune response. Since the primary innate immunity signaling pathways identified in *C. elegans* are highly conserved evolutionarily, the findings from the screen may also be extended to higher organisms, including humans. Nevertheless, although the nematode is a powerful tool to screen for antimicrobials, it cannot completely replace mammalian models and certain limitations must be acknowledged. Firstly, in the laboratory, *C. elegans* are routinely cultured at 16, 20, or 25°C. The worms cannot grow at 37°C and this restricts the range of pathogens that can be studied. Furthermore, *C. elegans* also has an efficient detoxification system, which limits the capacity to identify compounds that act via modulation of host defenses (Ewbank and Zugasti, 2011). That being said, the use of *C. elegans* in drug discovery still holds promise and should be pursued.

## Identification Of Novel Anti-Infectives

**FIGURE 1:**
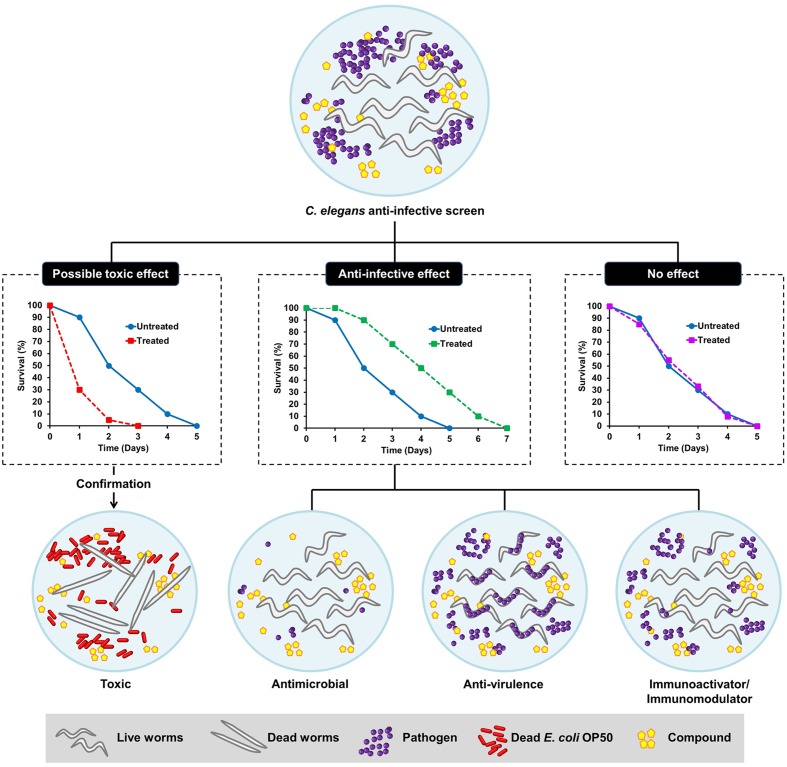
**Schematic diagram illustrating the possible outcomes of a *Caenorhabditis elegans*-based anti-infective screen.** From a screen, the compounds can be categorized as those with possible toxic effect, potential anti-infective effect or no effect. The hits that promote the survival of *C. elegans* following an infection can either act as anti-microbial, anti-virulence, or immunoactivator/immunomodulator. The toxic effect of a compound can be further validated by exposing the worms to the particular compound in an uninfected condition where the worms are fed with dead *Escherichia coli* OP50.

Generally, an anti-infective is an agent capable of preventing or counteracting infection, either by inhibiting the dissemination of an infectious agent or by killing the pathogen directly. Antibiotics are one of the well-known examples of anti-infective agents. Antimicrobial agents have been classified based on their ability to either kill the bacteria (bactericidal) or inhibit bacterial propagation (bacteriostatic) by targeting function essential to bacterial viability. Although these strategies are highly effective in eliminating the bacteria, they result in substantial stress on the target bacterium, leading to rapid selection of resistant sub-populations. Non-antimicrobial approaches to treat infections are therefore needed as alternative therapies to overcome the pitfalls of antibiotic resistance. Anti-infectives that do not affect microbial cell viability, but may instead interfere with virulence-mediated pathways in pathogens, appear as a new therapeutic paradigm (Allen et al., 2014). From the host perspective, selective modulation of the host innate immunity is another emerging concept driven by advances in the current understanding of host defense systems (Nijnik, 2013). Conventional *in vitro* cell-culture based assays are less likely to probe anti-infectives that target bacterial virulence or host immunity due to the absence of an intact and complex host–pathogen relationship. Following the wide application of *C. elegans* in infectious diseases modeling, this tiny nematode has emerged as a very useful model for anti-infectives drug discovery and development (Desalermos et al., 2011; Ewbank and Zugasti, 2011). In addition to its ability to identify novel antimicrobials that impair bacterial growth, the simple *C. elegans* model also presents an advantage, as we are able to select potential anti-virulence and immunomodulatory molecules in the context of a live animal. The possible outcomes of a *C. elegans*-based anti-infective screen are illustrated in **Figure 1**.

### Compounds with *In vivo* Antimicrobial Activity

In the first antimicrobial screen using *C. elegans*, the standard agar-based infection assay was modified to form a liquid-based curing assay in a 96-well plate format for high-throughput screening of 6,000 individually synthesized molecules and 1,136 natural extracts, consisting mainly of plant and marine extracts, for their ability to rescue *C. elegans* following an *Enterococcus faecalis* persistent infection (Moy et al., 2006). This screen identified 16 synthetic compounds and nine natural extracts that promote survival of *E. faecalis* infected worms. From the antimicrobial test, 7 of the 16 compounds inhibited the replication of *E. faecalis in vitro* with minimum inhibitory concentrations (MICs) <31 μg/mL. The screen was refined to a fully automated process which scaled up the throughput of the screen from ∼7000 compounds to ∼37,000 unique molecules and natural product extracts. This allowed the identification of 80 antimicrobial agents that cured *C. elegans* from a *E. faecalis* infection, including 62 known antibiotics and 18 candidates with previously reported antibacterial effect or structural analogs of known antibiotics (Moy et al., 2009).

The initial study by Moy et al. (2009) prompted other initiatives to search for novel anti-infectives and these efforts have been fruitful. To identify novel therapeutic molecules toward *S. aureus*, our group developed a *C. elegans*–*S. aureus* screening platform to perform small-scale *in vivo* screens on extracts from plant and marine resources as well as synthetic molecules (Kong et al., 2014b). In contrast to *E. faecalis* that persists in the *C. elegans* intestinal tract, *S. aureus* infection of worms is transient (Sifri et al., 2003). By constant exposure of *C. elegans* to *S. aureus* in liquid medium, 70 candidates were subjected to the *C. elegans*–*S. aureus* screen and 28 positive hits (14 extracts and 14 compounds) were obtained. Of the 14 natural extracts, 7 of them demonstrated potential antimicrobial effect against *S. aureus* when tested in a standard MIC microdilution assay. Among the most effective antimicrobial candidates were the extract from *Curcuma longa* and its main active component, curcumin (Kong et al., 2014b). An automated high-throughput *C. elegans* antimicrobial screen was used to screen a compound library comprising of 640 FDA-approved drugs for novel antimicrobial molecules against methicillin-resistant *S. aureus* (MRSA) (Rajamuthiah et al., 2014). Of note, closantel, the drug used to eradicate helminths, was identified as a promising hit that significantly prolonged the survival of MRSA-infected *C. elegans*. *In vitro* MIC microdilution tests showed that closantel exerted antibacterial activity against a wide range of Gram-positive and Gram-negative bacteria at low effective concentrations. Interestingly, closantel was also effective against the vancomycin-resistant *S. aureus* (VRSA) with an MIC of 0.78 μg/mL. The compound library also contained some clinically relevant antibiotics and almost all of the antibiotics that were previously known to be effective against MRSA were also identified as positive hits in the screen, further validating the robustness of the screen in detecting highly potential anti-staphylococcal molecules for future therapeutics development (Rajamuthiah et al., 2014). Following this, the screen has been expanded to two other compound libraries consisting of 85,000 compounds (Kim W. et al., 2015) and ∼21,000 compounds (Rajamuthiah et al., 2015), respectively. One positive hit from the screen was 1-Hexadecyl-2-methyl-3-(phenylmethyl)-1*H*-imidazolium iodide (NH125), an antibiotic with well-characterized mode of action (Yamamoto et al., 2000). A further study revealed that NH125 was also able to kill persister cells and eradicate the formation of MRSA biofilm by penetrating the extracellular polymeric substance (EPS) matrix within an established biofilm (Kim W. et al., 2015). Additionally, a novel antibacterial compound, 3-(phenylsulfonyl)-2-pyrazinecarbonitrile (PSPC), rescued MRSA-infected worms. PSPC and its derivatives displayed high antibacterial efficacy toward Gram-positive bacteria, with higher MIC values observed against Gram-negative pathogens (Rajamuthiah et al., 2015). Yang et al. (2013) developed a simple, time-efficient and cost-effective integrated microfluidic-based *C. elegans* screening system to test the *in vivo* antimicrobial effect of various natural compounds. The three major active components of the natural product rhubarb (emodin, rhein, and aloe-emodin) enhanced the survival of worms challenged with *S. aureus* and inhibited *S. aureus* replication at ≤16 μg/mL but were toxic to the animals at concentrations >60 μg/mL (Yang et al., 2013).

A *C. elegans*–*P. aeruginosa* liquid-based slow-killing assay was used to perform an *in vivo* screen for potential anti-infective substances against multidrug-resistant *P. aeruginosa*. Screening a collection of 1,300 natural extracts mainly consisting of secondary metabolites from fungal endophytes of medicinal plants, led to the discovery of four extracts from four different fungal species with both *in vivo* and *in vitro* antimicrobial activity (Zhou et al., 2011). The *in vivo* activity and efficacy of naturally occurring antimicrobial peptides isolated from frog skin were also evaluated using a *C. elegans* infection model (Uccelletti et al., 2010). The peptides, temporin-1Tb and esculentin (1–18), promoted the survival of *P. aeruginosa*-infected worms. Exposure to the peptides for 24 h significantly reduced the intestinal bacterial load in worms pre-infected with *P. aeruginosa*. Taking advantage of the transparent nature of *C. elegans*, the *in vivo* effect of the peptides on membrane permeability was examined by firstly exposing the *P. aeruginosa* infected nematodes to Sytox Green followed by treatment with the peptides and subsequently, observation of fluorescence intensity within the intestine of *C. elegans*. The enhanced fluorescence intensity in treated infected nematodes indicated that the peptide killed *P. aeruginosa in vivo* by permeating the bacterial membrane. The findings from this study reinforced the fact that *C. elegans* can be used to investigate the *in vivo* mode of action of antimicrobial molecules within the context of a living animal.

The high-throughput *C. elegans* screening platform has been expanded to identify potential antimicrobial therapeutics against other pathogens that cause clinically significant morbidity and mortality such as *Burkholderia pseudomallei* (Lakshmanan et al., 2014) and *A. baumannii* (Jayamani et al., 2015). Lakshmanan et al. (2014) described a robust *C. elegans* based high-throughput screen to search for novel antibiotics to treat melioidosis, a highly lethal infection caused by the Gram-negative *B. pseudomallei*. As *B. pseudomallei* is a Tier-1 organism, a closely related species, *Burkholderia thailandensis*, was substituted as the surrogate pathogen in the screen. One of the hits identified from the pilot screen of ∼30,000 compounds was Clinafloxacin, a fourth generation fluoroquinolone antibiotic, which demonstrated diverse antibacterial activity against *B. pseudomallei* as well as other Gram-negative pathogens (Lakshmanan et al., 2014). The ability of *A. baumannii* to infect and kill *C. elegans* led to the development of a *C. elegans*–*A. baumannii* liquid infection assay to screen for potential anti-*A. baumannii* agents from a collection of synthetic insect-derived antimicrobial peptides. A promising hit was the antimicrobial peptide, cecropin A, isolated from the mosquito *Aedes aegypti*. Further analysis suggested that this peptide exerted its antimicrobial activity by disrupting the bacterial membrane, leading to cell lysis (Jayamani et al., 2015). The *in vivo* antimicrobial effect of small molecules identified from an *in vitro* growth inhibition assay was evaluated using a *C. elegans*–*Burkholderia cenocepacia* infection model. Identification of a compound with good *in vitro* activity without curing *B. cenocepacia*-infected nematodes from infection highlights the importance of using a live animal model for the discovery of antimicrobial compounds with promising *in vivo* efficacy (Selin et al., 2015). Other drugs and natural compounds that have been tested for their *in vivo* antimicrobial properties in the worm model are listed in **Table 1**.

**Table 1:** Additional drugs/compounds evaluated for *in vivo* antimicrobial effects using a *Caenorhabditis elegans* model.

Drugs/compounds	Description	Infection model	Reference
Celecoxib	An FDA-approved non-steroidal anti-inflammatory drug that blocks the COX-2 enzyme. It is used to relieve fever, pain and inflammation.	*C. elegans* – MRSA	Thangamani et al., 2015
Lamotrigine	An FDA-approved anticonvulsant drug that also inhibits ribosome biogenesis in bacteria, thereby preventing the growth of bacteria.	*C. elegans–Salmonella enterica*	Stokes et al., 2015
Auranofin	A form of gold complex that is mainly implicated in the treatment of rheumatoid arthritis.	*C. elegans*–*S. aureus*	Fuchs et al., 2016
Artilysins	A modified form of endolysins, enzymes produced by bacteriophages to hydrolyze the bacterial cell wall, with improved outer membrane-penetrating capability.	*C. elegans–P. aeruginosa*	Briers et al., 2014
Berberine derivatives	Berberine is a natural alkaloid found in plants.	*C. elegans–E. faecalis C. elegans* – MRSA	Tomkiewicz et al., 2010 Dolla et al., 2015

### Compounds Targeting Bacterial Virulence

In the first antimicrobial screen by Moy et al. (2006) the identification of hits that did not interfere with bacterial growth *in vitro* or hits with a lower effective concentration *in vivo* suggests that these compounds and extracts may either act by enhancing host immunity or altering pathogen virulence. The findings from this study also offer proof-of-concept that the *C. elegans* whole-animal antimicrobial screening platform is able to detect hits that may be missed in a conventional cell-culture based *in vitro* screen where the live host–pathogen relationship is absent (Moy et al., 2006). In addition to novel antimicrobial compounds, the automated high-throughput *C. elegans* screen by Moy et al. also identified potential anti-virulence compounds (Moy et al., 2009), including analogs of small molecules that were found to prevent *P. aeruginosa* biofilm development in another anti-biofilm screen (Junker and Clardy, 2007).

In an earlier study on the effect of compounds on bacterial virulence, the effect of salicylic acid, a type of phenolic compound produced naturally by plants, was tested on *P. aeruginosa* virulence in the worm model as well as the *Arabidopsis thaliana* plant model. Feeding the worms with salicylic acid-treated *P. aeruginosa* prolonged worm survival compared to the worms exposed to untreated bacteria. Microarray and biochemical analyses proposed that this compound reduced the virulence of *P. aeruginosa* by inhibiting the production of biofilm, pyocyanin, protease, and elastase (Prithiviraj et al., 2005). Following this, the anti-virulence potential of extracts from three medicinal plants (*Conocarpus erectus, Callistemon viminalis*, and *Bucida buceras*) was assessed against *P. aeruginosa* using the *C. elegans* paralytic killing, fast-killing and slow-killing models. These extracts were known to affect bacterial quorum sensing (Adonizio et al., 2008a). Addition of the individual extracts into the solid agar medium significantly delayed the killing of nematodes by *P. aeruginosa* to a rate similar to worms fed with the *P. aeruginosa lasR* quorum sensing mutant, suggesting that these extracts most likely targeted the bacterial quorum sensing pathway (Adonizio et al., 2008b). A similar approach was also used to elucidate the anti-virulence properties of curcumin, the main bioactive component present in turmeric. Curcumin rescued *C. elegans* from a *P. aeruginosa* (PAO1) infection via the inhibition of various virulence factors, including pyocyanin, biofilm, acyl homoserine lactone, and bacterial quorum sensing (Rudrappa and Bais, 2008). In addition to its anti-virulence effect on *P. aeruginosa*, curcumin is also able to rescue worms from a *B. pseudomallei*-inflicted infection. We showed that curcumin interfered with *B. pseudomallei* iron acquisition and also inhibited the production of *B. pseudomallei* lipase, protease as well as biofilm without affecting bacterial viability. This suggests the potential of curcumin as an alternative therapy for the highly lethal melioidosis caused by *B. pseudomallei* (Eng and Nathan, 2015). In a recent study, curcumin was reported to interfere with bacterial virulence in a mouse model of lung infection, providing an independent validation of the anti-virulence effect identified in a *C. elegans* model (Wang et al., 2016).

Quorum sensing is a complicated cell-to-cell communication system that regulates the expression of various key virulence factors in Gram-positive and Gram-negative bacteria, making it a target of interest for anti-virulence therapy. The *P. aeruginosa* quorum sensing system utilizes acyl homoserine lactones as autoinducer molecules to regulate and direct the expression of virulence and biofilm-associated genes (Williams et al., 2007). Other natural inhibitors that have been tested for their anti-virulence effect *in vivo* using the *C. elegans*–*P. aeruginosa* infection model are the methanolic extract of *Terminalia chebula* fruit (Sarabhai et al., 2013), extract and active compound of *Dalbergia trichocarpa* bark (Rasamiravaka et al., 2015), tea polyphenols extracted from the leaf of *Camellia sinensis* L. (Yin et al., 2015), the methanolic extract of *Trigonella foenum-graecum* L. seed (Husain et al., 2015b), *Syzygium aromaticum* (clove) oil (Husain et al., 2013), and *Mentha piperita* (peppermint) oil (Husain et al., 2015a). In addition to targeting quorum sensing circuits, chemical compounds such as phenylacetic acid (Musthafa et al., 2012b), 2,5-piperazinedione (Musthafa et al., 2012a) and meta-bromo-thiolactone (O’Loughlin et al., 2013) as well as the hormonal therapy drug raloxifene (Ho Sui et al., 2012) inhibited *P. aeruginosa* pyocyanin production and reduced bacterial virulence in the nematode model. Other virulence determinants, including biofilm, proteases and elastase, as well as bacterial motility, were also suppressed by most of these molecules. Compounds targeting the iron-regulation pathway that alleviated *P. aeruginosa*-mediated killing of *C. elegans* were identified from a high-throughput liquid-based chemical screen (Kirienko et al., 2013). Recently, an anti-proliferative drug, 5-fluorouracil, was shown to enhance the survival of *P. aeruginosa*-infected *C. elegans* via disruption of pyoverdine biosynthesis (Kirienko et al., 2016). Interestingly, in an independent study, 5-fluorocytosine (a 5-fluorouracil precursor) demonstrated the ability to inhibit pyoverdine production *in vivo* in a mouse model of *P. aeruginosa* lung infection (Imperi et al., 2013). This compound also diminished *P. aeruginosa* virulence by targeting biofilm formation and quorum-sensing phenotypes *in vitro* (Ueda et al., 2009). Moreover, garlic extract also displayed anti-quorum sensing effect against *P. aeruginosa* in both nematode (Rasmussen et al., 2005) and mice models (Bjarnsholt et al., 2005; Harjai et al., 2010). Together, these findings demonstrate the successfully translation of the anti-virulence effect identified in a *C. elegans* model in a higher organism and provides a positive correlation between the compound activity identified in a *C. elegans* model with a more complex mammalian infection model. Through *in silico* drug design and model simulations, two chemical entities were identified as inhibitors of *P. aeruginosa* heme oxygenase. One of these compounds prolonged the lifespan of infected nematodes and decreased bacterial colony forming units in the *C. elegans* intestine, albeit showing poor antimicrobial activity *in vitro* (Hom et al., 2013). Recently, Zhu et al. (2015) used the *C. elegans* model to characterize an inhibitor of LasB, a vital virulence factor of *P. aeruginosa*. Cross-sectional transmission electron microscopy images of the *C. elegans* intestine displayed less host tissue damage upon treatment with the LasB inhibitor, suggesting that *P. aeruginosa* was rendered less virulent by the compound (Zhu et al., 2015).

*Staphylococcus aureus* is continuously acquiring resistance to clinically used antibiotics, underlining the need for new anti-virulence therapeutics against this pathogen. The ability of *S. aureus* to produce biofilm has complicated treatment by blocking the antibiotics penetration of the cells encased within the exopolysaccharide material. Hence, *S. aureus* biofilm and the quorum sensing system are attractive targets for anti-virulence therapeutics. Several natural compounds with *in vitro* anti-biofilm property have been demonstrated to attenuate *S. aureus* virulence *in vivo* in a *C. elegans* model. This includes essential oils from various plants (Lee J.H. et al., 2014; Lee et al., 2014a), red wine (Cho et al., 2015), stilbenes and the natural phenol, resveratrol (Lee J.H. et al., 2014; Lee et al., 2014b). Moreover, these natural products also reduced blood hemolysis caused by *S. aureus* hemolysins. The colonization of *S. aureus* in the worm intestine can be determined by staining the bacteria with acridine orange dye. Using this approach, the reduction of *in vivo* bacterial loads by an extract from a coral actinomycete was reported (Bakkiyaraj and Pandian, 2010). Hamamelitannin, the active component isolated from American witch hazel, has recently been shown to act as an inhibitor of the *S. aureus* quorum sensing mechanism. This compound and its analogs effectively improved the susceptibility of *S. aureus* biofilms to multiple antibiotics treatment both *in vitro* and *in vivo* (*C. elegans* and mouse infection models) (Brackman et al., 2016).

The uracil-auxotroph *E. coli* strain OP50 is routinely used as the laboratory-based food source for *C. elegans*, however, some pathogenic *E. coli* strains are lethal on worms. *E. coli* O157:H7 is a toxin-producing enterohemorrhagic strain with noteworthy medical implications. Broccoli extract and its associated flavonoid compounds were able to impede the virulence of this *E. coli* strain in the nematode model by inhibiting a number of vital virulence genes as well as the swarming motility of *E. coli* O157:H7 (Lee et al., 2011). Screening a collection of plant secondary metabolites for anti-biofilm activity against *E. coli* O157:H7 also identified several coumarin derivatives that were subsequently shown to suppress pathogen virulence *in vivo* in the *C. elegans* infection system (Lee J.H. et al., 2014). Lavigne et al. (2008) utilized the *C. elegans* model to compare virulence of *E. coli* strains isolated from urine samples of individuals who consumed either cranberry or placebo capsules. *E. coli* strains from patients who took regular cranberry capsules displayed reduced adherence *in vitro* and delayed killing of *C. elegans* (Lavigne et al., 2008). Apart from this, the *C. elegans* model has also been used to screen for anti-virulence compounds from a collection of ∼250 marine sponge associated bacterial extracts for their ability to rescue the nematodes from a *Vibrio alginolyticus* infection (Durai et al., 2013). The active ingredients isolated from plants and seawater bacteria inhibited *Vibrio cholerae* biofilm production and reduced *V. cholerae* virulence in the killing of nematodes (Kim H.I. et al., 2015; Rajalaxmi et al., 2016). Natural compounds that affected the virulence of Gram-positive *Clostridium difficile* (Yun et al., 2015) and *Listeria monocytogenes* (Silva et al., 2015; Sivaranjani et al., 2016) have also been recently described.

### Compounds Activating/Modulating Host Immune Responses

Despite lacking an adaptive immune system, *C. elegans* is still capable of activating protective mechanisms when confronted with pathogenic microorganisms in its natural habitat. The worms mount a complex and non-specific ancestral immune response, involving the activation of multiple signal transduction pathways and the secretion of immune effector molecules (Ermolaeva and Schumacher, 2014). This makes the identification of compounds targeting the host innate immune system possible using the *C. elegans*-based infection system. Following the initial high-throughput antimicrobial screen by Moy et al. (2009), the compound RPW-24 was shown to extend the lifespan of *P. aeruginosa*-infected worms without compromising bacterial integrity (Pukkila-Worley et al., 2012). By employing various approaches including microarray analysis using the readily available Affymetrix full-genome *C. elegans* GeneChip, as well as pathway elucidation studies using transgenic GFP reporter and mutant worms, the effect of the compound on host immunity was further characterized. RPW-24 was found to protect the worms from bacterial infection by inducing the p38 MAP kinase pathway-mediated response and the transcription factor ATF-7, suggesting the utility of the *C. elegans* model to discover novel strategies effective in treating bacterial infections (Pukkila-Worley et al., 2012). The extract from *Swietenia macrophylla* seeds promoted the survival of *P. aeruginosa*-challenged nematodes by boosting the expression of a *C. elegans* lysozyme encoding gene (*lys-7*) as observed by increasing fluorescence intensity in *lys-7*::GFP transgenic worms (Dharmalingam et al., 2012). Similarly, the nematodes subjected to *P. aeruginosa* infection were rescued by supplementation with dietary selenium. Selenite treatment did not interfere with bacterial quorum sensing and virulence, but elevated the transcript levels of host putative antimicrobial genes such as *lys-1*, *spp-1*, and *abf-1*. It was also demonstrated that the pathogen-resistant phenotype conferred by selenite requires the presence of a functional SKN-1 in *C. elegans* (Li et al., 2014).

From the *C. elegans*–*S. aureus* anti-infective screen discussed in Section “Compounds with *In vivo* Antimicrobial Activity,” our group identified an extract from a local plant, *Orthosiphon stamineus*, as a potential immunomodulatory drug that enhanced host tolerance toward a deadly *S. aureus* infection. This extract was not antimicrobial *per se* and is non-toxic to the *C. elegans* model. Expression of the host *lys-7* antimicrobial gene in untreated worms was suppressed after 24 and 48 h exposure to *S. aureus*. This suppression is mediated by the pathogen’s ability to alter the host defense mechanism, particularly the host antimicrobial response. As noted through observations of a fluorescing transgenic strain infected by *S. aureus*, the down-regulation of *lys-7* was restored in the presence of *O. stamineus* extract. Analysis using a loss-of-function *C. elegans* mutant established that the protective role of this extract is mediated via the conserved p38 MAPK and *daf-2/daf-16* insulin-like signaling pathways. qRT-PCR analysis of the host PMK-1 and DAF-16-regulated antimicrobial genes confirmed a significant positive modulation of these genes in *S. aureus*-infected worms exposed to *O. stamineus* extract. Further studies also revealed eupatorin as the major bioactive ingredient that contributed to the immunomodulation effect of *O. stamineus* (Kong et al., 2014a). In a separate study, *O. stamineus* extract also modulated the cellular immune response *in vitro* (Alshawsh et al., 2012). Similarly, the natural polyphenols isolated from Magnolia plant species, honokiol and magnolol, promoted a cellular immune response and slowed down *C. elegans* killing by *S. aureus* (Choi et al., 2015).

Host-directed immunomodulators offer a number of potential advantages over antimicrobial drugs. Targeting the host immunity in combating pathogen infection is an appealing approach as the stimulation or modulation of host immune function may promote resistance against a diverse array of pathogens, forming the basis of broad spectrum therapeutics to treat infections (Nijnik, 2013). Additionally, it may lower the risk of selection toward treatment resistance. The water-soluble cranberry extract was reported to up-regulate the expression of *C. elegans* innate immune genes and confer protection against various major pathogens, namely *V. cholerae*, *E. coli* O157:H7, *P. aeruginosa*, *Salmonella typhimurium*, *S. aureus*, and *E. faecalis* (Dinh et al., 2014). Specifically, the protective effect of this extract against *V. cholerae* is dependent on the p38 MAPK signaling pathway. The wide spectrum activity of other immunomodulatory compounds has also been seen for the alkaloid compounds harmane and colistin. Harmane promoted survival in nematodes infected by both Gram positive and Gram negative pathogens, including *E. coli* strain EDL933, *S. typhimurium*, *P. aeruginosa*, and *E. faecalis* (Jakobsen et al., 2013). Cai et al. (2014) developed a *C. elegans* – based chemical screen for the discovery of immune-inductive drugs using the *F35E12.5::gfp* transgenic strain and they identified the antibiotic colistin as a potential hit. The nematodes pre-treated with colistin were also resistant against *Yersinia pestis* and *P. aeruginosa* infections (Cai et al., 2014).

**FIGURE 2:**
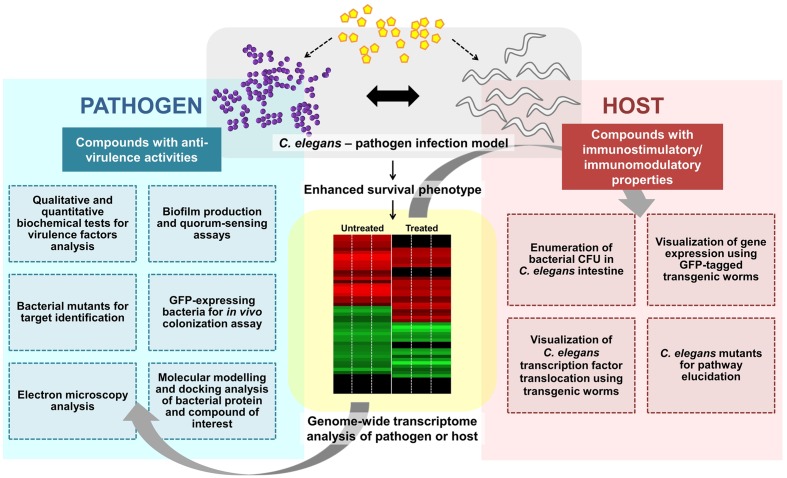
**Overview of the general experimental approaches used to characterize an anti-infective candidate.** A potential anti-infective compound that does not directly target bacterial growth can either impair pathogen virulence and/or enhance/modulate host immune responses. Various strategies from both pathogen and host perspectives can be employed to further characterize the molecular mechanism(s) of the compound of interest. Whole-genome transcriptome profiles of the compound-treated pathogen or host can be generated to study the effect of the compound. To assess the effect on bacterial virulence, qualitative and quantitative biochemical tests as well as biofilm production and quorum sensing assays can be performed. Bacterial mutants are useful for target identification while GFP-expressing bacteria may assist in visualization of *in vivo* bacterial colonization. Electron microscopy can be used to observe the formation of biofilm or bacterial structures (e.g., flagella). *In silico* molecular docking provides a clue of the possible binding interference between a bacterial protein and the compound of interest. From the host perspective, the live bacteria can be recovered from the *C. elegans* intestine and enumerated. The readily available transgenic mutant strains are useful for host target identification and pathway elucidation.

Concomitantly, some anti-infectives may exhibit both anti-virulence and immunomodulatory effects. The water extract of red seaweed *Chondrus crispus* exerted dual anti-infective effects by simultaneously disabling the virulence of *P. aeruginosa* and elevating host immunity to combat pathogen infection. *C. crispus* down-regulated mRNA levels of quorum sensing and virulence-related genes and retarded the secretion of *P. aeruginosa* virulence factors such as protease, elastase, pyocyanin, siderophore, hydrogen cyanide, and biofilm (Liu et al., 2013). Furthermore, the protective effect of *C. crispus* extract was diminished in selected *C. elegans* mutants (*pmk-1*, *daf-2*, *daf-16*, or *skn-1*), implying that the effect from the host perspective is mediated via the highly conserved immune pathways. Likewise, concurrent virulence inhibitory and immunostimulatory effects have also been observed in other seaweed-related natural products, including Tasco^®^, a commercially available animal feed supplement made from the brown seaweed *Ascophyllum nodosum* (Kandasamy et al., 2012) and another red seaweed species *Sarcodiotheca gaudichaudii* (Kulshreshtha et al., 2016). Both, *S. gaudichaudii* and *C. crispus* extracts disarmed the pathogenicity of *Salmonella enteritidis* by reducing biofilm formation, swimming and swarming motility and down-regulating expression of virulence and quorum sensing genes in *S. enteritidis.* These extracts also reduced the intestinal bacterial load in *C. elegans* by promoting the expression of host immune response genes encoding lectin-family, saponin-like and antibacterial proteins (Kulshreshtha et al., 2016). Whilst no direct evidence of seaweed extract exerting an immunomodulatory effect on animal infection-directed immune responses, is available, Luo et al. (2015) demonstrated that seaweed polysaccharide was immunomodulatory in a cell-culture based assay as well as in a mouse tumor model. The seaweed polysaccharide enhanced the tumor immune response by increasing the number of tumor-infiltrating immune-related cells in the murine lymphoma model. The various experimental procedures used to scrutinize the mode of action of a compound targeting bacterial virulence and/or host immunity are outlined in **Figure 2**.

Although, therapeutics that involve the immune system can be broad spectrum against a wide variety of infecting agents, overstimulation or dysregulation can lead to potential harmful side effects such as uncontrolled inflammatory response that may cause tissue damage or even worse, immunosuppression that may result in the shutdown of the entire immune system. For example, the immunomodulatory drug RPW-24 generally induced a *C. elegans* detoxification pathway and triggered *C. elegans* aversion behavior under an uninfected condition. Concurrently, the basal lifespan of the uninfected population was also shortened and the development of *C. elegans* larva was halted upon exposure to this compound (Pukkila-Worley et al., 2012). Therefore, the mechanism of immunostimulatory molecules must be carefully investigated to maximize the beneficial effect and at the same time minimizing the possible toxic effects. Furthermore, the precise mode of drug administration (e.g., prophylactic or during infection) should also be determined to ensure effective treatment.

## *C. elegans*: A Potential Repertoire Of Antimicrobial Proteins And Peptides (APPs)

In *C. elegans*, many of the pathogens are ingested and survive the passage through the worm grinder to establish an infection in the intestinal lumen, including *Serratia marcescens* (Schulenburg and Ewbank, 2004), *S. aureus* (Sifri et al., 2003), and *P. aeruginosa* (Tan et al., 1999). Certain pathogens can stably colonize the intestinal tract and eventually overwhelm the worm, leading to death. Such infections have been demonstrated for *S. typhimurium* (Aballay et al., 2000) and *E. faecalis* (Garsin et al., 2001). Similar to the intestine, but to a lesser extent, the epidermis that covers the worm external body surface is also vulnerable to pathogen attack. Some bacteria like *Microbacterium nematophilum* or *Leucobacter* spp. Verde adhere to the cuticle, causing swelling of the hypodermal tissue (Hodgkin et al., 2000, 2013) whilst the fungus *Drechmeria coniospora* adheres to the region of the mouth and vulva, and penetrates through the epidermis (Jansson, 1994). Yang et al. (2005) also demonstrated that the nematophagous fungus *Lecanicillium psalliotae* secretes serine protease that facilitates mycelia penetration and degradation of worm cuticle (Yang et al., 2005).

**FIGURE 3:**
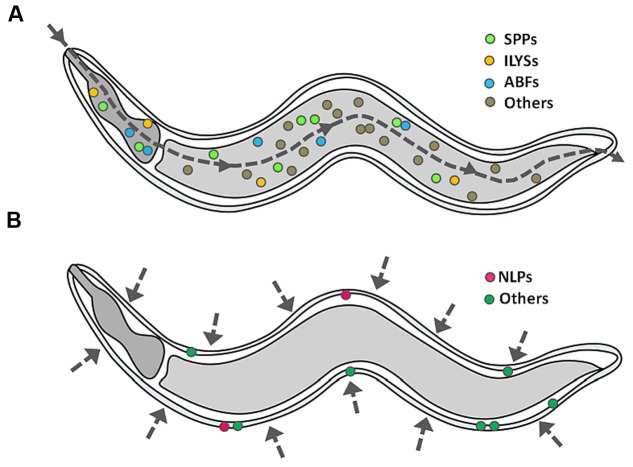
**Induction of APPs in *C. elegans*.** When *C. elegans* encounters a single pathogen, it will secrete a mixture of APPs as part of its protective mechanism, specifically **(A)** caenopores/saposin-like proteins (SPPs), antibacterial factor proteins (ABFs) and invertebrate lysozymes (ILYSs) in the digestive tract, and **(B)** neuropeptide-like proteins (NLPs) at the epidermis. The dashed line arrow denotes the route of infection.

With the aid of transcriptomics, it is now clear that infected worms are able to mount an inducible defense system, involving the activation of specific signaling pathways which lead to the release of immune molecules, including APPs (Couillault et al., 2004; O’Rourke et al., 2006; Shapira et al., 2006; Troemel et al., 2006; Zugasti and Ewbank, 2009; Gravato-Nobre et al., 2016). As opposed to conventional antibiotics, many APPs directly act on the membrane of the pathogen and thus, the development of microbial resistance by gene mutation is less likely (Peschel and Sahl, 2006). In some cases, APPs may possess immunomodulatory functions (Lai and Gallo, 2009; Nijnik and Hancock, 2009; Afacan et al., 2012), including (i) reduction in the levels of pro-inflammatory cytokines, (ii) modulation of the expression of chemokines, reactive oxygen species and reactive nitrogen cytokines, (iii) stimulation of angiogenesis, (iv) wound healing, and (v) leukocyte and macrophage differentiation. Up till now, a diverse array of putative APPs has been identified based on their induced expression upon infection or sequence similarities (Kato et al., 2002; Pujol et al., 2008), however, how these different APPs act cooperatively has not yet been characterized. For this review, we only focus on the representative families of APPs that have been documented to exhibit *in vitro* antimicrobial activity at the peptide/protein level. **Figure 3** depicts the tissue-specific induction of APPs in *C. elegans*.

### Caenopores

Examination of the worm’s genome led to the discovery of saposin-like peptides/proteins (SPPs) characterized by an α-helix bundle stabilized by three unique disulphide bonds (Bruhn, 2005). Alternatively, they are named as caenopores owing to their structural and functional resemblance with amoebapores as they consist of a secretory signal peptide followed by saposin-like domain (Roeder et al., 2010). Given that most *spp* gene expression is regulated by the intestine specific transcription factor ELT-2, it is likely that caenopores are exclusively expressed in the intestine (Hoeckendorf and Leippe, 2012; Hoeckendorf et al., 2012). The direct role for caenopores in the innate immune response of *C. elegans* was first reported in Banyai and Patthy (1998) when the antibacterial effect of recombinant SPP-1 was observed on *E. coli*. Like amoebapores, SPP-5 was shown to display pore-forming activity and kill bacteria by permeabilising the cytoplasmic membrane (Roeder et al., 2010). A detailed examination of three-dimensional structure revealed that SPP-5 has five amphiphatic helices, connected by three disulfide bonds arranged like a folded leaf, which is the signature of the SAPLIP family (Mysliwy et al., 2010). It was worth noting that this was the first and only representative structure solved for *C. elegans* APPs. The molecular mechanism underlying pore formation however remains unclear.

### Antibacterial Factor (ABF) Peptides/Proteins

A total of six ASABF (*Ascaris suum* antibacterial factor) homologs or best known as antibacterial factor (ABF-1 to ABF-6) peptides/proteins have been identified in *C. elegans*. ASABF is a cysteine-rich microbicidal factor that was first discovered in the body fluid of the nematode *A. suum* (Kato and Komatsu, 1996). It is possible that ABFs are also part of the *C. elegans* defense mechanism as exposure to pathogens led to the up-regulation of *abf* genes (Alegado et al., 2003; Alper et al., 2007; Means et al., 2009). By means of RNAi-mediated gene silencing, the functional significance of ABFs was further characterized. Silencing one of these genes, *abf-2*, led to increased bacterial load in the intestinal lumen (Alegado et al., 2003). It is well-established that expression of *abf* genes is localized primarily at the site of contact with bacteria, i.e., in the digestive tract (Alper et al., 2007). To date, only recombinant ABF-2 is known to display broad-spectrum antimicrobial activity, with the greatest effect observed toward Gram-positive bacteria (Kato et al., 2002). The precise role(s) of other members of this family are still unclear and required further analysis.

### Invertebrate Lysozymes (ILYS)

*Caenorhabditis elegans* harbors protist-type (*lys-1* to *lys-10*) and invertebrate type (*ilys-1* to *ilys-6*) lysozyme encoding genes (Schulenburg and Boehnisch, 2008; Tarr, 2012). Representative members of the latter are up-regulated upon exposure to various pathogen insults (Irazoqui et al., 2010), suggesting possible roles involved in host defense. Studies using recombinant ILYS-3 have reported that the protein displays lytic activity against the Gram-positive *Micrococcus luteus* and *M. nematophilum*. It is known that invertebrate lysozymes are able to cleave the isopeptide bonds established between D-glutamate and L-Lysine present in peptidoglycans (Tarr, 2012; Van Herreweghe and Michiels, 2012). The importance of invertebrate lysozymes for host defense was recently confirmed when Gravato-Nobre et al. (2016) reported that disruption of *ilys-3* renders the mutant worms more susceptible to *M. nematophilum*. The investigators also elegantly showed that ILYS-3 is primarily produced in the *C. elegans* digestive tract (Gravato-Nobre et al., 2016). For additional information of the protist-type lysozymes, readers are referred to previous reviews (Schulenburg and Boehnisch, 2008; Ewbank and Zugasti, 2011).

### Neuropeptide-Like Peptides/Proteins (NLPs)

In *C. elegans*, most of the neuropeptide-like peptides/proteins *(nlp)* genes were named as such because of their limited sequence similarity with YGGXamide neuropeptide genes, sharing the YGGWG and YGGYG motifs (Nathoo et al., 2001). These genes form “the *nlp-29* cluster” which comprises *nlp-27* to *nlp-31* and the adjacent gene *nlp-34* on chromosome V (Couillault et al., 2004; Pujol et al., 2008). Upon exposure to the nematode-trapping fungus *Monacrosporium haptotylum*, several *nlp* genes were induced in infected worms (Fekete et al., 2008). Overexpression of these genes enhanced worm resistance to *D. coniospora* infection, further validating their contribution to *in vivo* host defense (Pujol et al., 2008). Couillault et al. (2004) demonstrated that the 53-amino-acid synthetic NLP-31 has potent antimicrobial activity against fungi and several bacteria. Utilizing transgenic worms expressing GFP under the control of the *nlp-31* promoter, these investigators also demonstrated that this gene is exclusively expressed in the hypodermis. Recent work from our group has proposed an immunomodulatory role for NLP-31 as the peptide regulated the expression of inflammatory cytokines in *B. pseudomallei*-infected macrophage cells. Several lines of evidence suggest that it employs a mechanism that does not involve membrane permeabilisation, but instead interact with cytoplasmic macromolecules to interfere with the viability of *B. pseudomallei* (Lim et al., 2016).

## Concluding Remarks

Active research on the development of anti-infective therapeutics that target bacterial virulence and/or host immune response using a *C. elegans* host model indicates a paradigm shift in the understanding of the host–pathogen interface. The existence of notable similarities at the molecular and cellular levels between nematode and higher vertebrates opens a path toward the discovery of novel therapeutics for human infections that might exhibit superior function over conventional antibiotics. The findings generated in the simple nematode model provide insights into the modulation of bacterial virulence and host immunity to fight infectious diseases and can readily be translated to higher organisms. Despite numerous efforts to tackle the spread of antimicrobial resistance, bacteria continue to show reduced susceptibility toward antibiotics over time and the rate of new drug discovery is declining rapidly. Developing alternative anti-infectives that do not affect bacterial cell viability hold great potential to compensate for this significant health care challenge. In addition, *C. elegans* APPs also provide a parallel opportunity to uncover host candidates for drug discovery.

## Author Contribution

All authors listed, have made substantial, direct and intellectual contribution to the work, and approved it for publication.

## Conflict of Interest Statement

The authors declare that the research was conducted in the absence of any commercial or financial relationships that could be construed as a potential conflict of interest.
